# NAFLD and NASH in HCV Infection: Prevalence and Significance in Hepatic and Extrahepatic Manifestations

**DOI:** 10.3390/ijms17060803

**Published:** 2016-05-25

**Authors:** Luigi Elio Adinolfi, Luca Rinaldi, Barbara Guerrera, Luciano Restivo, Aldo Marrone, Mauro Giordano, Rosa Zampino

**Affiliations:** Department of Medical, Surgical, Neurological, Metabolic, and Geriatric Sciences, Second University of Naples, Naples 80100, Italy; lucarinaldi@hotmail.it (L.R.); barbara.guerrera@alice.it (B.G.); luciano.restivo@gmail.it (L.R.); Aldo.marrone@unina2.it (A.M.); mauro.giordano@unina2.it (M.G.); rosa.zampino@unina2.it (R.Z.)

**Keywords:** HCV-associated NAFLD, insulin resistance, liver fibrosis, HCC, metabolic syndrome, diabetes, atherosclerosis

## Abstract

The aim of this paper is to review and up to date the prevalence of hepatitis C virus (HCV)-associated non-alcoholic fatty liver disease (NAFLD) and non-alcoholic steatohepatitis (NASH) and their significance in both accelerating progression of HCV-related liver disease and development of HCV-associated extrahepatic diseases. The reported mean prevalence of HCV-related NAFLD was 55%, whereas NASH was reported in 4%–10% of cases. HCV genotype 3 directly induces fatty liver deposition, namely “viral steatosis” and it is associated with the highest prevalence and degree of severity, whereas, HCV non-3 genotype infection showed lower prevalence of steatosis, which is associated with metabolic factors and insulin resistance. The host’s genetic background predisposes him or her to the development of steatosis. HCV’s impairment of lipid and glucose metabolism causes fatty liver accumulation; this seems to be a viral strategy to optimize its life cycle. Irrespective of insulin resistance, HCV-associated NAFLD, in a degree-dependent manner, contributes towards accelerating the liver fibrosis progression and development of hepatocellular carcinoma by inducing liver inflammation and oxidative stress. Furthermore, NAFLD is associated with the presence of metabolic syndrome, type 2 diabetes, and atherosclerosis. In addition, HCV-related “metabolic steatosis” impairs the response rate to interferon-based treatment, whereas it seems that “viral steatosis” may harm the response rate to new oral direct antiviral agents. In conclusion, a high prevalence of NAFLD occurs in HCV infections, which is, at least in part, induced by the virus, and that NAFLD significantly impacts progression of the liver disease, therapeutic response, and some extrahepatic diseases.

## 1. Introduction

Non-alcoholic fatty liver disease (NAFLD) is a condition characterized by fatty liver accumulation with a spectrum of liver damage ranging from simple steatosis to non-alcoholic steatohepatitis (NASH). The latter accounted for one third of cases [1] and it is a common cause of chronic liver diseases, including cirrhosis and hepatocellular carcinoma (HCC) [2]. NAFLD is strictly associated with metabolic syndrome in the general population and can be considered as a multisystem disease associated with inflammation, oxidative stress, and insulin resistance with an increasing risk of type 2 diabetes mellitus, cardiovascular diseases, and chronic kidney diseases [1]. Moreover, irrespective of metabolic syndrome, recently, several host genetic backgrounds have been reported as potential risk factors for development of NAFLD [1].

NAFLD is a prominent feature of chronic hepatitis C virus (HCV) infection [3]. Both viral and host factors contribute to the development of steatosis. NAFLD in HCV genotype 3 infected patients is strictly associated with serum viral load [3,4,5,6,7], thus steatosis in this setting is considered to be of viral origin and it is namely “viral steatosis”; whereas in HCV non-3 genotype infected patients, NAFLD is mainly linked to host factors such as body mass index (BMI), obesity, particular visceral obesity [8], insulin resistance, and type 2 diabetes mellitus, and it is called “metabolic steatosis”. Accordingly, liver steatosis localization in HCV non-3 genotypes infected patients is similar to that observed in NAFLD/NASH (*i.e.*, mostly in the centrolobular zone (acinar 3)) [9], whereas in genotype 3 infection steatosis is localized mainly in the periportal zone (acinar 1) [10]. With respect to the sustained virologic response rate to interferon-based treatment, a substantial difference in the behavior of the two types of HCV-associated NAFLD has been reported. Metabolic steatosis significantly reduces response rate to interferons [6,11,12,13,14], whereas virologic steatosis does not impact the interferon response rate and it even disappears following HCV clearance with reappearance in relapse cases [5,15]. In addition, HCV-related metabolic steatosis is strictly associated with insulin resistance; although HCV *per sé* induces insulin resistance, which predates the development of steatosis, that, in turn, aggravates insulin resistance [16,17]. Furthermore, it has been demonstrated that HCV-associated steatosis induces hepatic and systemic inflammation and oxidative stress [18,19].

**Figure 1:**
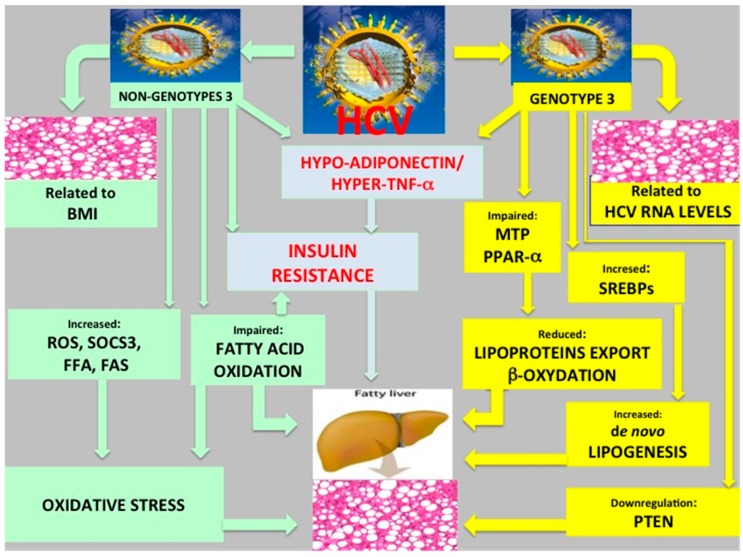
Schematically are illustrated the mains HCV genotype-specific molecular mechanisms of steatogenesis. Abbreviations used: HCV: hepatitis C virus; BMI: body mass index; MTP: microsomal triglyceride transfer protein; PPAR-α: peroxisome proliferator-activated receptor α; SREBPs: sterol regulatory element-binding proteins; ROS: reactive oxygen species; SOCS3: suppressor of cytokine signaling 3; FFA: free fatty acid; FAS: fatty acid synthase; PTEN: phosphatase and tensin homolog.

The mechanisms by which HCV induces steatosis are complex and specific for genotype 3 (viral steatosis) and non-3 genotypes (metabolic steatosis). However, the two forms of steatosis share some mechanisms and overlapping conditions may occur. Recently, we reviewed the main molecular mechanisms by which HCV induces steatosis [20] and in Figure 1 the chief genotype-specific mechanisms are reported.

Chronic HCV infection is considered a systemic disease and there is evidence that steatosis may change the natural history of both HCV-related hepatic and extrahepatic diseases, and that host genetic backgrounds may promote HCV-associated steatosis and progression of liver disease.

In this paper we reviewed the prevalence and associated factors which promote NAFLD/NASH in chronic HCV infections and the evidence that highlights the role of steatosis in both accelerating the progression of HCV-related liver disease and the development of HCV-associated extrahepatic diseases.

## 2. Prevalence of NAFLD/NASH and Associated Conditions in Chronic HCV Infection

In chronic HCV infection, NAFLD has been reported with a mean prevalence of about 55%, ranging from 40% to 86% [3,5,6,21,22,23,24,25,26,27,28,29,30] depending on HCV genotype and local prevalence of metabolic syndrome. HCV genotype 3 infected patients showed the highest prevalence of steatosis (up to 86%), whereas in HCV genotype 1 and 2 the mean reported prevalence was about 40% and 50%, respectively. The above reported prevalence of NAFLD-associated to HCV infection is higher than the rates observed in non-HCV infected subjects in the general population (*i.e.*, 20%–30%) [31], and of the rates reported for other hepatic diseases, such as HBV infection (about 22%) and autoimmune hepatitis (about 16%) [17].

Data on the prevalence of NASH in chronic hepatitis C cases are less consistent than those reported for NAFLD. The published data reported an occurrence of NASH from 4% to 10% [25,32,33,34,35]. Risk factors associated with development of HCV-associated NASH include BMI and, for HCV genotype 1, triglyceride and HDL-cholesterol levels, whereas for genotype 3, aspartate transaminase levels are a contributing factor [35,36].

A strict association between HCV-associated NAFLD and insulin resistance has been reported. However, insulin resistance can be both a direct consequence of HCV infection and a result of NAFLD, and *vice versa* [17]. Overall, insulin resistance has been reported with high prevalence in chronic hepatitis C infection cases (up to 80%) and it is commonly observed in HCV non-3 genotypes infected patients, whereas it is not a feature of HCV genotype 3 infection [19].

Overweight and obese BMI levels significantly contribute to the development of HCV-associated NAFLD, in particular, it has been demonstrated that visceral obesity has a preeminent role [2]. Visceral obesity plays an important role in the regulation of glucose and lipid metabolism in chronic HCV infection. In HCV-infected patients with visceral obesity, it has been reported that there are increased levels of pro-inflammatory cytokines (IL-6 and TNF-α) that inhibit insulin signaling and the secretion of adiponectin, which results in corresponding consequences for the development of liver steatosis and insulin resistance [37]. The latter represents the pathophysiological link between steatosis and the metabolic syndrome. Despite chronic HCV patients showing a high prevalence of insulin resistance, an overall low prevalence of full-blown metabolic syndrome was reported [38]. Patients with HCV-associated steatosis presented a higher prevalence of metabolic syndrome than those without steatosis, but a lower prevalence than that observed in NAFLD patients [38]. However, HCV infection is associated with multiple metabolic derangements, which has been termed hepatitis C associated dysmetabolic syndrome (HCADS) [20]. Such metabolic derangements are characterized by insulin resistance, hypocholesterolemia, hyperuricemia, and altered body fat distribution [20].

It has been demonstrated that oxidative stress occurs with high prevalence in chronic HCV infection (e.g., greater that 60% [18]), and that it contributes to the development of NAFLD in HCV non-3 genotypes, but not in “viral steatosis” associated with HCV genotype 3 infections.

The host’s genetic background has an important impact on the development of NAFLD in chronic HCV infections. It has been demonstrated that microsomal triglyceride transfer protein (MTP) polymorphism (493GT) was associated with a higher prevalence of NAFLD in HCV genotype 3 [39]; methylene tetrahydrofolate reductase (MTHFR) polymorphism (C677T) [40] was correlated with an increased prevalence of NAFLD in chronic HCV infections as well as an increased risk to develop severe steatosis (*i.e.*, 6-fold higher for hosts with a MTHFR “CT” genotype and 20-fold higher for those with a “TT” genotype) [40]. The patatin-like phospholipase domain-containing 3 (PNPLA3) gene, in particular, its I148M variant, has been linked with an increased prevalence of HCV-related NAFLD and with visceral obesity [41,42]. Recently, we demonstrated that the TM6SF2, E167K variant, contributes to liver steatosis in chronic hepatitis C [43].

## 3. HCV-Induces Steatosis: Is It a Finalistic Condition?

A characteristic feature of HCV infections is the strict association between viral factors and host metabolic factors (*i.e*., lipid and glucose metabolism), which are involved in the development of liver steatosis. Experimental and clinical evidence showed that HCV core proteins, in a genotype-specific manner, cause hepatic fat accumulation by activating SREBP-1 and 2 [44], inhibiting MTP activity [45], impairing peroxisome proliferator-activated receptor (PPAR) expression, and promoting de novo lipid synthesis [46], which harms assembly, excretion, and uptake of very low density lipoprotein (VLDL).

The interaction between HCV and the host’s lipid metabolism seems to be crucial for the viral life cycle. It is reported that triglyceride-rich VLDL represents an essential role in the assembly and secretion of HCV. Elements of infective HCV circulate in patient sera as lipo-viro particles (LVPs) in association with ApoB- and ApoE-containing lipoproteins, which suggests the association of viral particles with LDL and VLDL. It was reported that the interaction with the LDL receptor is important for HCV entry into hepatocytes [47]. Similarly, synthesis of farnesyl pyrophosphate and geranylgeranyl pyrophosphate are essential for HCV replication [48]. On the bases of this evidence it has been hypothesized that the abnormalities of hepatic lipid content are essential to perpetuate the HCV life cycle [49].

## 4. HCV-Associated Steatosis and Progression of Liver Damage

One important question was to define if NAFLD/NASH could impact hepatic fibrosis progression in chronic hepatitis C infections through modifying the natural history of liver damage. Earlier cross-sectional studies demonstrated an association between NAFLD and advanced liver fibrosis [3,8] as well as an association between NAFLD and liver inflammation, which was also strictly associated with progression of fibrosis [50]. Such data were confirmed by prospective studies using paired liver biopsies. A study by Westin *et al.* [25] that featured paired liver biopsies for98 HCV patients showed that steatosis, especially in genotype 3, was an independent factor associated with fibrosis progression. Similarly, Castera *et al.* [28] evaluated the fibrosis progression in 96 chronic hepatitis C patients by means of paired liver biopsy with a mean interval of four years, and found that steatosis was an independent factor associated with fibrosis progression via performing a multivariate analysis (odds ratio (OR) = 4.7%–95% CI = 1.3–10.8; *p* = 0.0001). In addition, Cross *et al.* [51] also used multivariate analysis tin a study involving 112 chronic hepatitis C patients with serial liver biopsy to show that fibrosis progression was associated with steatosis (OR: 14.3; 95% CI: 2.1–1110; *p* = 0.006).

Twenty-eight other cross-sectional or prospective studies, carefully reviewed by Lonardo *et al.* [20], evaluated the association between steatosis and fibrosis confirming that steatosis is strictly associated with liver fibrosis in chronic HCV infections. However, there were some studies that reported an association between steatosis and liver fibrosis that was genotype-dependent [7,25,29,51,52].

A meta-analysis, including data from 10 centers in Europe, Australia, and North America for 3068 individuals with chronic hepatitis C, analyzed the independent factors associated with liver fibrosis [53]. The meta-analysis showed that steatosis was independently associated with liver fibrosis (OR: 1.66; 1.27–2.18: *p* < 0.001) and with liver inflammation. The data of the meta-analysis also reinforce the hypothesis that inflammation is the link between steatosis and liver fibrosis progression.

It is important to underline that there were a marginal number of studies that were not able to demonstrate an association between liver fibrosis and steatosis [22,29,53,54,55,56]. The discrepancy of such results may be explained by differences in study design, patient demographic characteristics, differences in histological grading of steatosis/fibrosis, type of statistical analysis performed, and confounding variables, in particular, insulin resistance which has been reported to be independently associated with both steatosis and liver fibrosis progression [53,57,58,59]. Overall, the majority of the studies evaluated the role of steatosis without considering insulin resistance or *vice versa*, due to the overlapping conditions, thus their independent role in the progression of liver fibrosis has not yet been adequately assessed. However, Moucari *et al.* [60] evaluated 500 patients with chronic hepatitis C and the multivariate analysis showed that both steatosis (adjusted OD 1.95, 1.24–3.06, *p* = 0.004) and insulin resistance (adjusted OD: 1.80, 1.15–2.81, *p* = 0.009) were independently associated with advanced liver fibrosis. Hu *et al.* [61], in a retrospective study including 460 patients with chronic hepatitis C, also showed that grade 2 and 3 levels of steatosis were independently associated with liver fibrosis.

**Figure 2:**
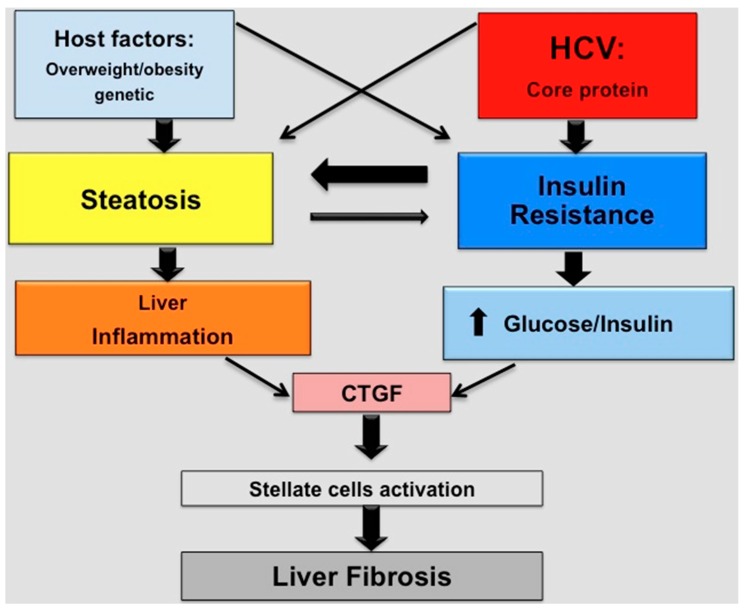
Schematic representation of factors and mechanisms involved in the progression of liver fibrosis in chronic hepatitis C patients. HCV: hepatitis C virus: CTGF: connective tissue growth factor.

The mechanisms by which steatosis and insulin resistance induce progression of liver fibrosis seem to be different. On the basis of the data within the literature [17,52,62,63], in Figure 2 we schematically reported such mechanisms. Both steatosis and insulin resistance activate connective tissue growth factor (CTGF), but steatosis does so by increasing inflammation [17,53] while insulin resistance does so by increasing glucose and insulin levels [62,63].

Fartoux *et al.* [64] showed that the cumulative probability of progression of fibrosis in mild chronic hepatitis C during a follow up period of more than 90 months was strictly associated with the presence of steatosis. In Fartoux’s study, patients with less than 5% steatosis showed a negligible progression of liver fibrosis; patients with steatosis greater than 30% showed the highest (and statistically significant) progression of fibrosis (*p* < 0.0001), and patients with steatosis between 5% and 30% showed an intermediate progression of liver fibrosis.

It has been reported that similar to patients with HCV-associated NAFLD, those with NASH showed advanced fibrosis [32]. The data seem to suggest that the biological significance of NASH-associated with HCV infection is similar to that observed for cases with a high-degree of steatosis.

In conclusion, there is significant evidence that steatosis is strictly associated with faster progression of liver fibrosis in chronic hepatitis C. The fibrogenic effect of HCV-associated steatosis seems to be multi-factorial involving pro-inflammatory cytokines, oxidative stress, insulin resistance, glucose levels, and increased susceptibility to apoptosis.

There is experimental and clinical evidence supporting a role of HCV-related steatosis in the development of HCC. In experimental models, using transgenic mice, HCV core proteins showed a causative role in the development of steatosis and HCC [65,66,67,68]. The experimental evidence has been confirmed in the majority of clinical studies performed [67,69,70,71,72,73,74,75,76]. Both retrospective and prospective studies, with only a few exceptions, showed that HCV-related steatosis was strictly associated with the development of HCC and that the amount of fatty liver deposition was an important risk factor for HCC [67,69,70,71,72,73,74,75,76]. Thus, HCV patients with the highest degree of steatosis carry a higher risk of HCC. Accordingly, HCV genotype 3 infection has been reported to convey the highest risk to develop HCC [67]. However, at present, direct evidence supporting a role for viral steatosis in inducing HCC is lacking. It has also been shown that patients with HCV-related steatosis and diabetes have an enhanced risk of HCC [77]. The data underline the necessity to increase surveillance for HCC in patients with HCV-related steatosis and advanced liver disease.

It seems that HCV-related steatosis may influence the development of HCC by several mechanisms. Among these, it has been suggested that oxidative stress may have a role through reactive oxygen species inducing mutagenesis [78] and both insulin resistance and lipid metabolic alterations are considered hepato-carcinogenic factors in HCV-related steatosis [79].

## 5. HCV-Associated Steatosis, Diabetes, Metabolic Syndrome, and Atherosclerosis

HCV infection is associated with an increased risk of type 2 diabetes [80]; HCV patients showed a 12 times higher risk to develop type 2 diabetes [81]. Similarly, diabetes patients had a 5–10 times increased risk of being HCV positive [82]. It has been demonstrated that, in predisposed individuals, chronic HCV infection accelerates the appearance of type 2 diabetes by at least 10 years [81]. The mechanism involved in HCV-induced diabetes is insulin resistance, which is strictly associated with both viral infection and steatosis. It has been reported that 75% of HCV patients with diabetes have steatosis [83] and that HCV-related steatosis is associated with diabetes [53]. Thus, HCV-related steatosis may influence diabetes by aggravating insulin resistance. Otherwise, it is also possible that both insulin resistance and type 2 diabetes can increase or aggravate steatosis in chronic HCV infection.

The presence of steatosis, insulin resistance, and diabetes are associated with advanced liver fibrosis, HCC, and poor outcome of chronic hepatitis C infections [84,85,86].

The prevalence of metabolic syndrome in chronic hepatitis C patients was about 5% and was similar to that observed in the general population [37]. However, metabolic syndrome was significantly higher in patients with HCV-related steatosis as compared with HCV patients without steatosis (13.3% *vs.* 1.8%) [50]. The data indicate that in chronic hepatitis C patients the presence of liver steatosis predicts metabolic syndrome.

There is a consistent body of literature demonstrating that chronic HCV infection is a risk factor for atherosclerosis, cardiovascular diseases, and related forms of mortality [18]. It has been demonstrated that HCV may live and replicate within carotid plaque [87]. Moreover, chronic HCV infection is associated with many pro-atherogenic conditions such as inflammation, hypoadiponectinemia, hyperhomocysteinemia, increased oxidative stress, insulin resistance, and diabetes [18]. HCV-related steatosis was associated with the above reported pro-atherogenic conditions [50]. Thus, it was anticipated that hepatic steatosis might predict the presence of atherosclerosis in chronic hepatitis C patients. Accordingly, it has been demonstrated that in HCV patients, steatosis is an independent factor associated with the highest prevalence of atherosclerosis [50]. Steatosis predicted, with a good specificity (81.7%) and sensitivity (74.2%), both early (intima-media thickness) and advanced (plaques) lesions of carotid atherososclerosis. It has been suggested that steatosis may modulate atherogenic factors; such as inflammation and metabolic elements favoring the development of atherosclerosis and that patients with HCV-associated steatosis should be screened for atherosclerosis [50].

## 6. HCV-Associated Steatosis and Response to Antiviral Treatments

HCV-related “metabolic steatosis” has been reported as a negative predictor of response to interferon-based antiviral therapy in genotypes 1 and 2 infections [6,11,12,13,14]. In this setting of treatment, patients with “viral steatosis” associated with HCV genotype 3 infection were considered easy to treat.

In the last few years, the new oral direct antiviral agents (DAAs) are becoming the standard of HCV treatment. The impact of steatosis on DAAs treatment has been scantly evaluated. However, there is an agreement that the HCV genotype 3 showed lower response rate to DAAs and now it has been considered to be difficult to treat. It has been proposed that steatosis could partly explain the lower response rate in HCV genotype 3 infections [88]. Thus, specific studies are needed to evaluate the impact of steatosis and metabolic factors on the response rate of the new DAAs.

## 7. Conclusions

The data demonstrate that hepatic steatosis is a feature of chronic HCV infections and that liver fatty accumulation seems to be a finalistic condition favoring the persistence and replication of HCV. HCV-associated steatosis, in a degree-dependent fashion, producing hepatic inflammation and oxidative stress, induces a more rapid progression of liver fibrosis and increases the risk of the development of HCC. HCV-associated steatosis also influences the development of some extrahepatic manifestations of chronic HCV infection such as diabetes, metabolic syndrome, and atherosclerosis. In addition, the presence of steatosis impairs the response rate to interferons based anti-HCV treatments and could have a role in the lower response rate observed in HCV genotype 3 treated with new DAAs. Thus, steatosis should be regarded as a marker to individuate patients at higher risk of progression of HCV-associated liver disease, development of extrahepatic diseases, and lower therapeutic response rate, perhaps even in the era of new DAAs.
